# Ningxiang pigderived *Enterococcus hirae* regulates the inflammatory function and enhances the protection of piglets against ETEC challenge

**DOI:** 10.3389/fcimb.2024.1476564

**Published:** 2024-10-17

**Authors:** Longlin Zhang, Zichen Wu, Zihao Zhang, Rong Cai, Shujun Pang, Jing Wang, Xiyuan Bao

**Affiliations:** ^1^ College of Animal Science and Technology, Hunan Agricultural University, Key Laboratory for Quality Regulation of Livestock and Poultry Products of Hunan Province, Changsha, China; ^2^ Yuelushan Laboratory, Changsha, China; ^3^ Department of General Surgery, No. 924 Hospital of Joint Logistics Support Force of PLA, Guilin, China

**Keywords:** ETEC, piglets, inflammatory cytokines, spleen, acetate

## Abstract

This study investigated the effects of *Enterococcus hirae* (Eh) derived from Ningxiang pigs on growth performance, diarrhea incidence, and immune responses in ETEC-challenged piglets. The results showed that compared to the CON group, ETEC infection significantly reduced the average daily gain (ADG) and average daily feed intake (ADFI), increased rectal temperature, and resulted in a diarrhea rate of up to 24%. Additionally, ETEC infection significantly increased the spleen index and the expression of inflammatory cytokines in the spleen, serum and intestine, with decreasing serum sIgA and colonic SCFAs of piglets. Compared to the ETEC group, orally Eh significantly increased ADFI in ETEC-infected piglets, reduced the diarrhea rate to 11.53%, reduced the spleen index and the expression of inflammatory cytokines in the spleen, serum and intestine, with decreasing serum sIgA and colonic SCFAs of ETEC-infected piglets. Furthermore, correlation analysis revealed that the levels of SCFAs (particularly acetate) were significantly negatively correlated with the expression levels of inflammatory cytokines in colonic and splenic tissues, suggesting that acetate may be a key metabolite in the anti-inflammatory effects of Eh. These results indicate that Eh can enhance the protection of piglets against ETEC K88 via intestine-acetate-spleen axis, thereby alleviating diarrhea and improving growth performance in piglets.

## 1 Introduction

ETEC infections, particularly those caused by the K88 serotype, are a leading cause of diarrheal diseases in animals, leading to severe intestinal infections and related illnesses (Vidal et al., 2016; Sun and Kim, 2017; Yue et al., 2020; Zhang et al., 2022a). The intestine is not only crucial for nutrient digestion and absorption but also serves as the largest immune organ. ETEC K88 infection disrupts this vital system by inducing intestinal inflammation and compromising the intestinal barrier through bacterial attachment and enterotoxin secretion, which can trigger a systemic inflammatory response in the host (Zhang et al., 2022a). Extensive evidence indicates that ETEC K88 infection adversely affects growth and increases mortality in livestock, causing significant economic losses to the livestock industry (Augustino et al., 2022; Ntakiyisumba et al., 2022). Historically, antibiotics have been extensively used as growth promoters and antimicrobial agents in animals (Rahman et al., 2022). However, the rise of antibiotic-resistant bacteria and the banning of antibiotics in feed have shifted research focus towards finding viable alternatives to antibiotics to combat the challenges posed by ETEC K88 infections.

Lactic acid bacteria hold promise as potential alternatives to antibiotics due to their anti-inflammatory and antimicrobial properties and their ability to modulate the intestinal microbiota (Ayivi et al., 2020; Abedin et al., 2023). Zeyner et al. found that administering *E. faecalis* daily from birth to weaning reduced diarrhea rates (Zeyner and Boldt, 2006). Strompfova et al. reported that administering *E. faecalis* EK13 to neonatal piglets significantly reduced fecal staphylococcus counts on day 1. After 7 days of feeding, *E. faecalis* EK13 significantly reduced fecal *E. coli* counts (Strompfová et al., 2006). Additionally, *E. faecium* T-013 and other probiotic composites have been shown to improve the intestinal environment of pigs, reduce NH3 emissions, enhance the breeding environment, and boost the immune system (Franz et al., 2003).

As a novel strain of lactic acid bacteria, *Enterococcus hirae* (Eh) has been recognized as a potential probiotic in human diseases and food (Goubet et al., 2021; Li et al., 2024). Our previous study found that Eh HNAU0516, isolated from Ningxiang pigs with high resistance, could promote the intestinal development of weaned piglets, enhance intestinal barrier function, and improve gut microbiota (Zhang et al., 2024). Furthermore, it significantly inhibited *E. coli* activity in an *in vitro* bacteriostatic assay, suggesting its potential as an antibiotic alternative. Therefore, the present study utilized an ETEC K88-infected piglet model to validate the hypothesis that Ningxiang pig-derived Eh HNAU0516 could attenuate ETEC K88-induced inflammatory responses and intestinal health dysfunction.

## 2 Materials and methods

All experiments were conducted in accordance with the guidelines of the Institutional Animal Care and Use Committee of Hunan Agricultural University.

### 2.1 Bacterial strain

The Eh HNAU0516 strain used in this study was initially isolated from feces of healthy Ningxiang piglets and conserved in the China Center for Type Culture Collection (CCTCC NO.M20221530, Wuhan, Hubei, China). Eh HNAU0516 was cultured in MRS medium (No. HB0384-1, Qingdao) for 12 h at 37°C. The counts of viable probiotic bacteria in the Eh -containing supplements were verified via a cultural method using a MRS agar medium (No. HB0384, Qingdao). ETEC k88 was kindly provided by Prof. Wenkai Ren, from South China Agricultural University (Guangzhou, China). And the counts of viable bacteria in the ETEC -containing supplements were verified via cultural method using a LB agar medium (No. HB0128, Qingdao) for 12 h at 37°C.

### 2.2 IPEC-J2 cells

The IPEC-J2 cells were maintained in DMEM/F12 medium (BI, Dibosi Biological Technology, Co., Ltd., Shanghai, China), supplemented with 100 U/mL penicillin, 100 μg/mL streptomycin, and 10% (vol/vol) heat-inactivated fetal bovine serum (FBS) (BS-1101, Inner Mongolia Opcel Biotechnology Co.,Ltd., Neimenggu, China), at 37°C and 5% CO_2_ in a humidified incubator. Cells (10^6^ cells per well) were plated in 6-well plates and co-cultured with live Eh (10^6^ CFU) for 2h and ETEC (10^5^ CFU for 1 h).

### 2.3 Animals and experimental design

**Figure 1:**
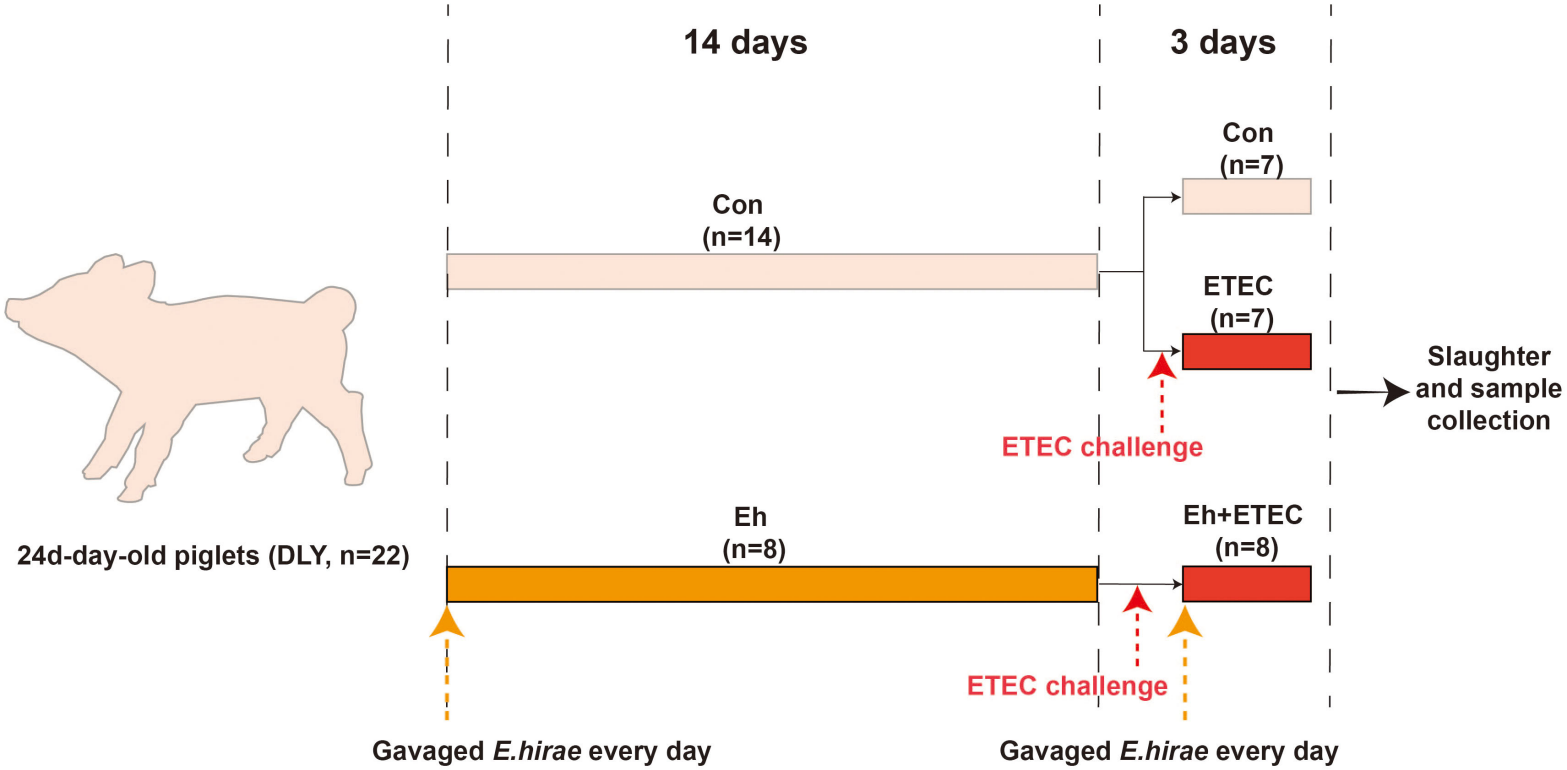
Experimental design diagram. From 0 to 14 d, CON group received a standard diet and daily gavage of 10 mL of sterile saline; Eh group received a standard diet and gavage of 10^9^ CFU/mL (10 mL) *Enterococcus hirae (E.hirae*) every day. At 15 d, all groups except the CON group received a daily gastric infusion of 10^9^ CFU/mL (10 mL) ETEC, and the CON group received a gastric infusion of sterile saline using the same method. From 15 to 17 d, Eh+ETEC group continue received a standard diet and gavage of 10^9^ CFU/mL (10 mL) *E.hirae* every day, while the CON group and ETEC group received a gastric infusion of sterile saline using the same method.

Twenty-two healthy Duroc × Landrace × Yorkshire (DLY) piglets, aged 24 days, were randomly divided into different group with similar body weights (BWs). From 0 to 14 d, Con group received a standard diet and daily gavage of 10 mL of sterile saline; Eh group received a standard diet and gavage of 10^9^ CFU/mL (10 mL) Eh every day. At 15 d, all groups except the CON group received a daily gastric infusion of 10^9^ CFU/mL (10 mL) ETEC, and the Con group received a gastric infusion of sterile saline using the same method. From 15 to 17 d, Eh+ETEC group continue received a standard diet and gavage of 10^9^ CFU/mL (10 mL) Eh every day, while the CON group and ETEC group received a gastric infusion of sterile saline using the same method. Each consisting of 7 or 8 replicates with 1 piglet per replicate. No antibiotics were given to the animals throughout the trial for prophylactic or therapeutic reasons. All piglets had free access to feeding and drinking water. Room temperature was maintained at approximately 27-30 °C, and the humidity was controlled between 50%-60%. Piglets were sacrificed by exsanguination after electrical stunning, and serum, intestine and fecal samples were collected for further analysis. The experimental design diagram is shown in **Figure 1**. The basic formula and nutritional level are presented in **Supplementary Table S1**.

### 2.4 Diarrhea rate, and relative organ weight

Visceral organs (liver and spleen) were removed and weighed. The relative weight of each organ = organ weight/final live body weight. Additionally, the fecal score (0, normal; 1, mild diarrhea; 2, medium diarrhea; 3, serious diarrhea) of each piglet was recorded daily and their diarrhea rate were also be calculated. Diarrhea rate was calculated as a percentage of the number of diarrheal piglets during the period divided by the total number of piglets (day × piglets).

### 2.5 Histopathology and mucus staining

For histologic analysis, the duodenum, jejunum and colon (mid-section) tissues were fixed in 10% formalin for 24 hours at room temperature, embedded in paraffin, and sectioned at a thickness of 3 mm for staining. As previously reported (Zhang et al., 2021), 10-μm-thick sections were stained with hematoxylin and eosin and analyzed under a microscope (BX53, Olympus, Tokyo, Japan). Histological analysis was performed using ImageJ software. Additionally, AB-PAS staining was performed to verify the function of mucous-secreting goblet cells. Sections (10-μm) from the tissue were stained with AB (Abcam, Cambridge, UK) and PAS (Millipore Sigma, Billerica, MA, USA) staining kits according to the manufacturer’s protocol. It is also visualized using an Olympus microscope (BX53, Olympus, Tokyo, Japan).

### 2.6 Total RNA extraction and real-time reverse transcription PCR

Total RNA was prepared using the RNeasy Kit (SENO Biotech Co., Ltd., Zhangjiakou, China). cDNA was synthesized from total RNA using the SuperScript IV First Strand Synthesis System (AG11711, Accurate Biotechnology Co., Ltd, Changsha, China) according to the manufacturer’s instructions. A 7500 Fast Real-Time PCR system (Applied Biosystems, Foster City, CA, USA) was used for quantitative PCR analysis. The primers used are listed in **Supplementary Table S2**.

### 2.7 Serum inflammatory biomarkers

Serums were centrifuged for 90 s at 15,000 g and serum aliquots were snap-frozen until further analysis. Quantification of cytokine levels in serum was assessed using a commercially available porcine cytokine multiplex immunoassay kit (RayBiotech, Norcross, GA). And the concentration of cytokines was calculated by QAP-CYT-1 software. Additionally, the sIgA concentrations were determined by ELISA assay (Ruixinbio Quanzhou, China). Briefly, serum was prepared according to the standard procedures. The enzyme-linked reaction was carried out according to the commercial kit, and the absorbance value at 450 nm was finally determined. Concentrations of sIgA were calculated from the standard curve.

### 2.8 SCFAs analysis

The concentrations of SCFAs including acetate, propionate, butyrate and valerate were analyzed using the gas chromatographic method. Briefly, as previously reported (Zhang et al., 2024), 1.0 g of colonic chyme was first fully homogenized with 1.5 mL of deionized water. The above fecal homogenate was centrifuged at 15 000 × g for 10 min at 4°C. The samples were acidified with 25% metaphosphoric acid at a ratio of 1:5 for 30 min on ice. Samples were injected into a gas chromatographic 8890 series gas chromatograph (Agilent, USA) for detection.

### 2.9 Statistical analysis

The data were analyzed using SPSS 26.0 statistical software (ver. 26.0 for Windows, SPSS Inc., Chicago, IL, USA). The diarrhea rates of piglets were compared with use of chi-square analysis. For the continuous variables with a normal distribution, one-way ANOVA was used to analyze the difference among groups, and the homogeneity of variance among groups was further tested using LSD multiple-range tests. And correlation test between the inflammatory biomarkers and SCFAs was explored by R package. And P-value < 0.05 was considered statistically significant, 0.05 ≤ P-value < 0.1 was considered statistically trend. All data were expressed as means with their standard errors.

## 3 Results

### 3.1 Growth performance

As showed in **Table 1**, there were no significant differences in the average daily gain (ADG), average daily feed intake (ADFI) or the ratio of feed to gain (F/G) among two group from days 1 to 14 of the experimental period. During days 15-17, ETEC challenge significantly decreased ADG and ADFI compared with CON group. Furthermore, the ADFI was numerically higher in Eh+ETEC group than in the ETEC group, with the ADG was no significant differences in two groups (**Table 2**). Additionally, there were no significant differences in the F/G among three groups (**Table 2**).

**Table 1:** Growth performance of piglets in each group before ETEC challenge.

Items	CON	Eh	*p*-value
ADG, kg/d	0.32 ± 0.028	0.30 ± 0.021	0.697
ADFI, kg/d	0.40 ± 0.020	0.37 ± 0.025	0.410
F/G, kg/kg	1.33 ± 0.185	1.29 ± 0.024	0.421

Data are presented as the Means ± SEM. ADG, average daily gain. ADFI, average daily feed intake. F/G, average daily feed intake to average daily gain.

**Table 2:** Growth performance of piglets in each group after ETEC challenge.

Items	CON	ETEC	Eh+ETEC	*p*-value
ADG, kg/d	0.30 ± 0.032^a^	0.16 ± 0.028^b^	0.22 ± 0.026^ab^	0.017
ADFI, kg/d	0.44 ± 0.024^a^	0.26 ± 0.040^b^	0.42 ± 0.028^a^	0.002
F/G, kg/kg	1.39 ± 0.163	1.73 ± 0.226	1.79 ± 0.213	0.238

a,b In the same row, different superscript letters indicate significant differences (p < 0.05), and the same superscript letters indicate no significant difference (p > 0.05). Data are presented as the Means ± SEM. ADG, average daily gain. ADFI, average daily feed intake. F/G, average daily feed intake to average daily gain.

### 3.2 Diarrhea rate and rectal temperature

During days 1-14, there was no difference in the diarrhea rate between the Eh and CON groups (7.14% vs. 3.57%) (**Supplementary Figure 1A**). During days 15-17, although there was no significant difference in diarrhea rates, the ETEC group had the highest rate (CON vs. ETEC vs. Eh+ETEC: 8.00% vs. 24.00% vs. 11.53%) (**Supplementary Figure 1B**). Meanwhile, the rectal temperature of piglets was significantly increased after the ETEC challenge (**Supplementary Figure 1C**).

### 3.3 Viscera index and spleen inflammatory expression

**Figure 2:**
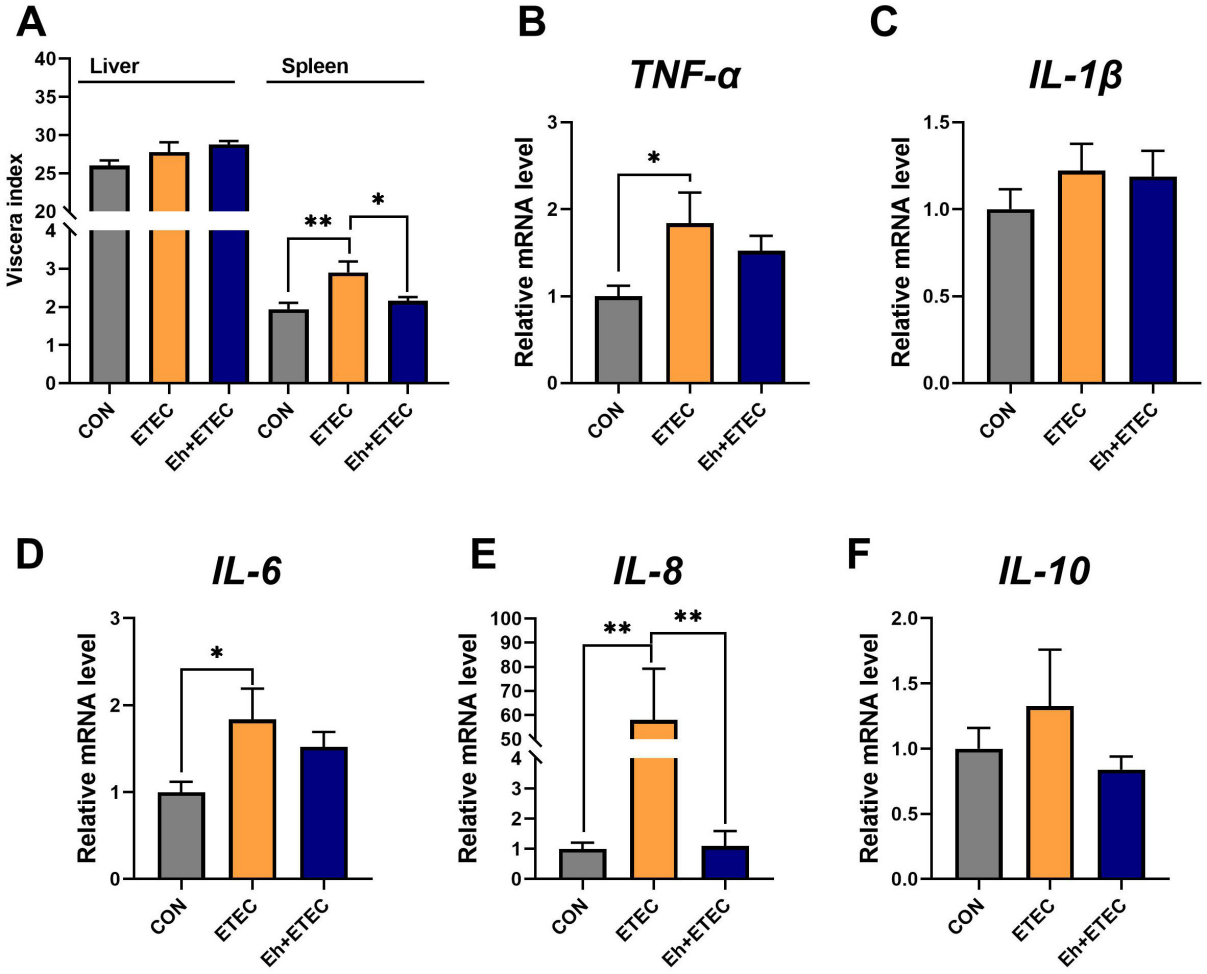
Effect of *E.hirae* on spleen inflammatory expression in ETEC-challenge piglets. **(A)** The viscera index of piglets, including liver and spleen. **(B–F)** The effects of *Enterococcus hirae* in spleen TNF-α **(B)**, IL-1β **(C)**, IL-6 **(D)**, IL-8 **(E)** and IL-10 **(F)**. Mean ± SEM are shown. * 0.01 ≤ p ≤ 0.05; **0.001 < p ≤ 0.01. CON group, ETEC group, n=7; Eh+ETEC, n=8.

Eh significantly reduced the spleen index in ETEC-challenged piglets, while there was no difference in the liver index (**Figure 2A**). Compared to the CON group, ETEC challenges significantly increased the levels of TNF-α, IL-6, and IL-8 in the spleen (**Figures 2B, D, E**). Oral administration of Eh significantly decreased the level of IL-8 in ETEC-challenged piglets (**Figure 2E**), while there was no difference in TNF-α and IL-6 levels (**Figures 2B, D**). Additionally, there was no difference in the levels of IL-1β and IL-10 in the spleen among the three groups (**Figures 2C, F**).

### 3.4 Serum inflammatory expression

**Figure 3:**
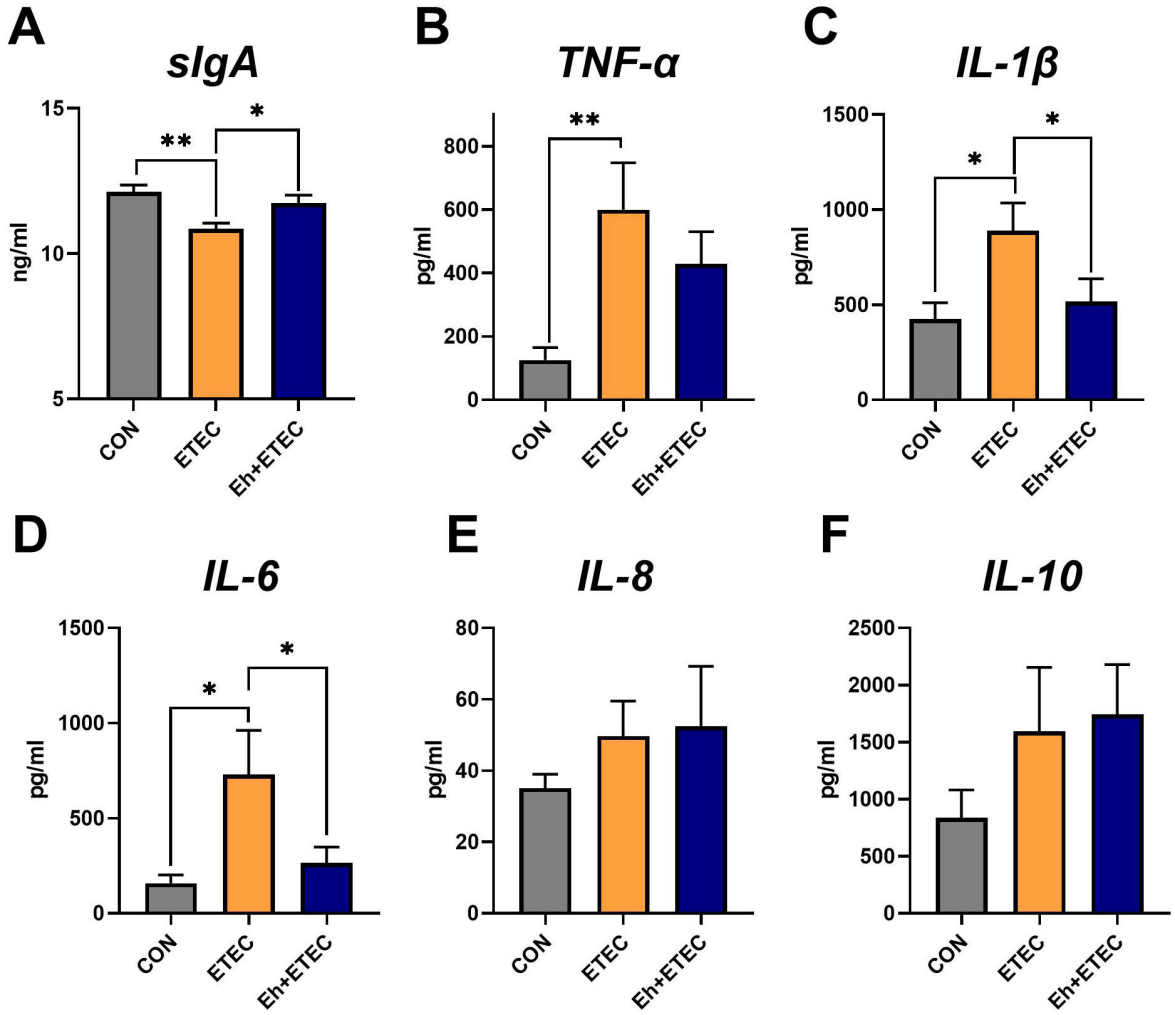
Effects of *Enterococcus hirae* on serum inflammatory expression in ETEC-challenged Piglets. **(A)** The sIgA levels in serum. **(B–F)** The effects of *Enterococcus hirae* in serum TNF-α **(B)**, IL-1β **(C)**, IL-6 **(D)**, IL-8 **(E)** and IL-10 **(F)**. Mean ± SEM are shown. * 0.01 ≤ p ≤ 0.05; **0.001 < p ≤ 0.01. CON group, ETEC group, n=7; Eh+ETEC, n=8.

Additionally, ETEC challenges significantly decreased the levels of serum sIgA, and oral administration of Eh reversed this effect (**Figure 3A**). Compared with the CON group, ETEC challenges significantly increased the levels of TNF-α, IL-1β, and IL-6 in serum (**Figures 3B–D**). Oral administration of Eh significantly decreased the levels of IL-1β and IL-6 in ETEC-challenged piglets (**Figures 3C, D**), while there was no difference in TNF-α levels (**Figure 3B**). Additionally, there was no difference in the levels of IL-8 and IL-10 in serum among the three groups (**Figures 3E, F**).

### 3.5 Intestine morphology and intestinal inflammatory expression

**Figure 4:**
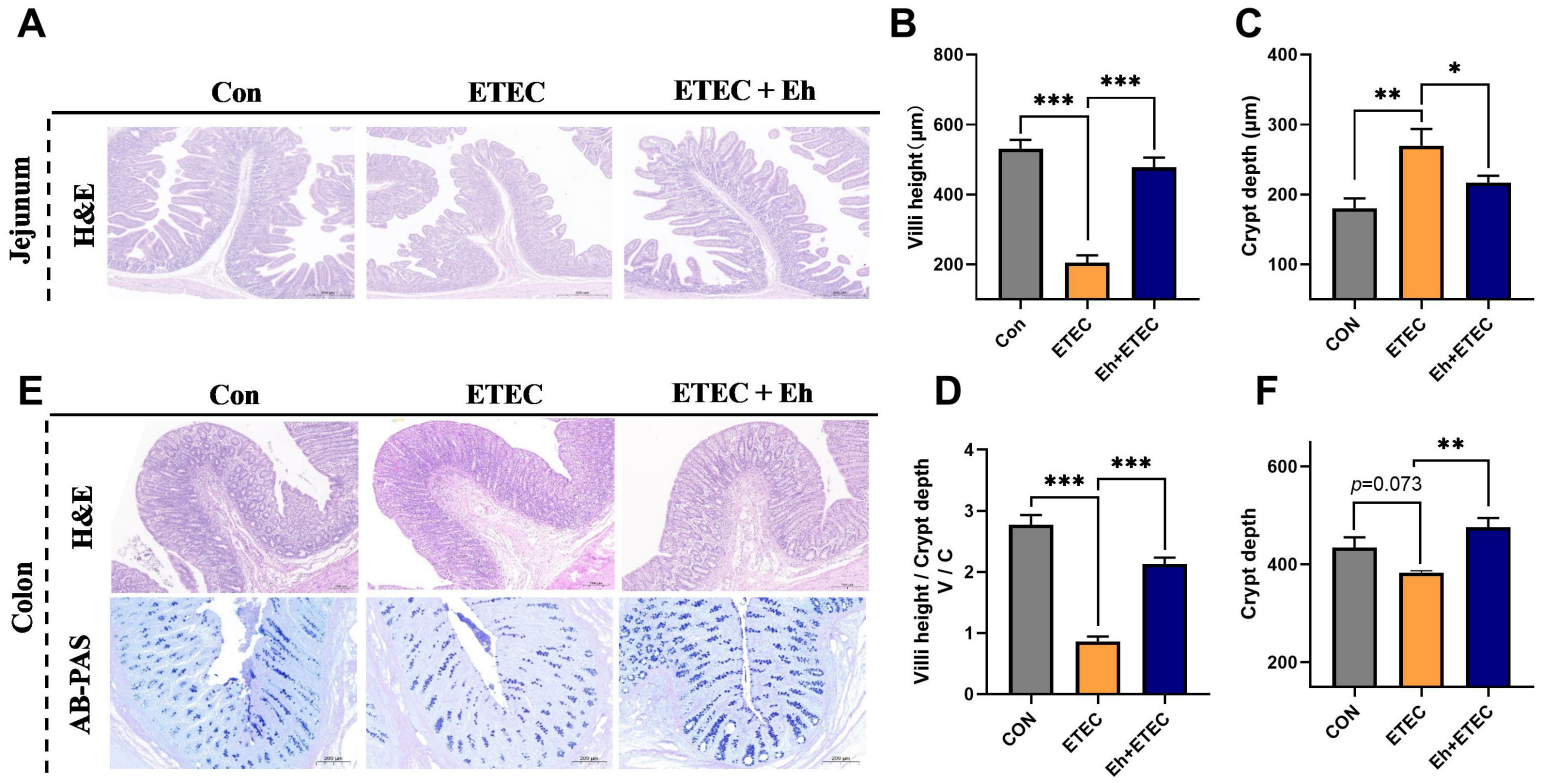
Effects of *Enterococcus hirae* on intestinal morphology in ETEC-challenged Piglets. **(A)** The H&E representative images of the jejunum. **(B–D)** The villi height **(B)**, crypt depth **(C)** and villi height/crypt depth (V/C) **(D)** of jejunum. **(E)** The H&E and AB-PAS representative images of the colon. **(F)** The crypt depth of colon. * 0.01 ≤ p ≤ 0.05; **0.001 < p ≤ 0.01; *** p ≤ 0.001. Mean ± SEM are shown. CON group, ETEC group, n=7; Eh+ETEC, n=8.

As shown in **Figure 4**, jejunum and colon morphology were examined using H&E staining to determine the effects of Eh treatment on ETEC-challenged piglets. In the jejunum, compared with the CON group, ETEC challenges significantly decreased villi height and V/C, and increased crypt depth, whereas oral Eh increased villi height and V/C, and decreased crypt depth in ETEC-challenged piglets (**Figures 4A–D**). In the colon, ETEC challenges showed a trend of decreasing crypt depth, whereas oral Eh increased crypt depth in ETEC-challenged piglets (**Figures 4D, E**). Additionally, AB-PAS staining was used to show colonic goblet cell density. Compared with the CON group, ETEC challenges decreased the number of goblet cells, whereas oral Eh increased the number of goblet cells in ETEC-challenged piglets (**Figure 4D**).

In the jejunum, compared with the CON group, ETEC challenges significantly increased the expression levels of TNF-α and IL-6, while oral administration of Eh significantly decreased these inflammatory factors (**Table 3**). In the colon, compared with the CON group, ETEC challenges significantly increased the expression levels of TNF-α, IL-1β, IL-6, and IL-8, while oral administration of Eh significantly decreased these inflammatory factors (**Table 2**). Furthermore, the IPEC-J2 cell model was used to investigate the effect of Eh on the expression of inflammation challenged by ETEC (**Supplementary Figure S2A**). Compared with the CON group, ETEC challenges significantly increased the expression levels of TNF-α, IL-1β, IL-6, and IL-8 and decreased the expression levels of IL-10, while administration of Eh significantly reversed these inflammatory factors (**Supplementary Figure S2B**).

**Table 3:** Effect of *E.hirae* on the intestinal inflammatory expression in ETEC-challenge piglets.

Items	CON	ETEC	Eh+ETEC	*p*-value
Jejunum				
*TNF-α*	1.00 ± 0.17^b^	2.307 ± 0.48^a^	1.0999 ± 0.19^b^	0.034
*IL-1β*	1.00 ± 0.26	2.5825 ± 1.09	1.1772 ± 0.16	0.256
*IL-6*	1.00 ± 0.10^b^	2.3338 ± 0.57^a^	0.8475 ± 0.17^a^	0.024
*IL-8*	1.00 ± 0.23	1.7406 ± 0.48	1.2177 ± 0.23	0.374
*IL-10*	1.00 ± 0.19	1.5388 ± 0.35	0.8681 ± 0.12	0.149
Colon				
*TNF-α*	1.00 ± 0.07^b^	1.98 ± 0.27^a^	0.96 ± 0.15^b^	0.002
*IL-1β*	1.00 ± 0.10^b^	1.66 ± 0.21^a^	0.53 ± 0.08^c^	0.000
*IL-6*	1.00 ± 0.04^b^	1.97 ± 0.40^a^	1.44 ± 0.14^ab^	0.067
*IL-8*	1.00 ± 0.09^b^	2.10 ± 0.39^a^	1.12 ± 0.24^b^	0.042
*IL-10*	1.00 ± 0.13	1.22 ± 0.07	1.24 ± 0.15	0.553

a,b,c In the same row, different superscript letters indicate significant differences (p < 0.05), and the same superscript letters indicate no significant difference (p > 0.05). Data are presented as the Means ± SEM.

### 3.6 SCFAs

**Figure 5:**
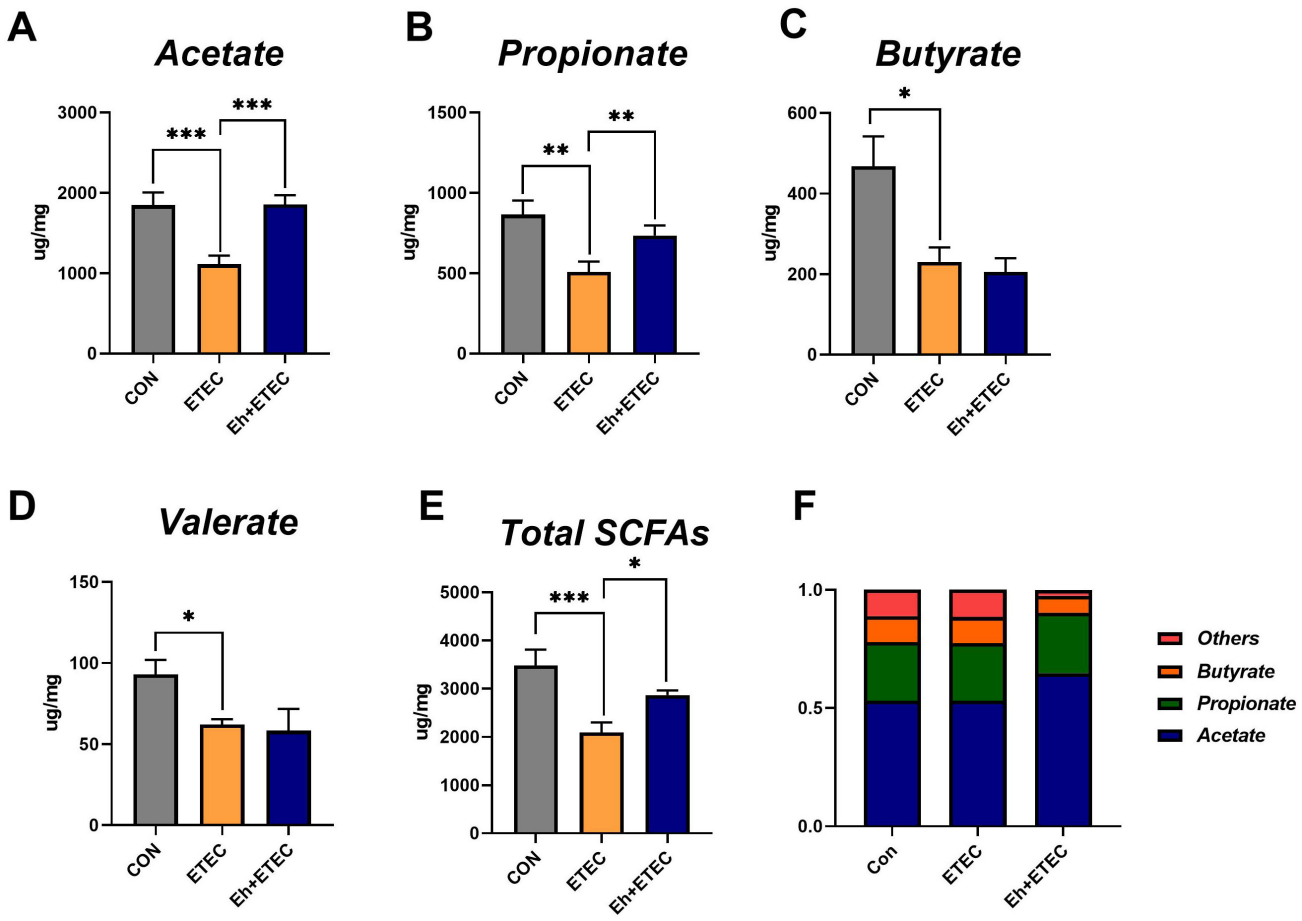
Effects of *Enterococcus hirae* on SCFAs in ETEC-challenged Piglets. **(A–E)** The concentration of acetate **(A)**, propionate **(B)**, butyrate **(C)**, valerate **(D)**, and total SCFAs **(E)** in colonic contents of piglets. **(F)** The relative content of each SCFA in colonic contents of piglets. The Mean ± SEM are shown. * 0.01 ≤ p ≤ 0.05; **0.001 < p ≤ 0.01; *** p ≤ 0.001. CON group, ETEC group, n=7; Eh+ETEC, n=8.

As shown in **Figure 5**, colonic short-chain fatty acids were measured. The results showed that ETEC challenges significantly decreased the concentrations of acetate, propionate, butyrate, valerate, and total SCFAs, while oral administration of Eh significantly increased the concentrations of acetate, propionate, and total SCFAs in ETEC-challenged piglets (**Figures 5A–E**). The ratio of acetate, propionate, butyrate, and valerate in total SCFAs was calculated and shown in **Figure 5F**, suggesting that oral administration of Eh resulted in a higher proportion of acetate in ETEC-challenged piglets.

### 3.7 Correlation of SCFAs levels with inflammatory biomarker

**Figure 6:**
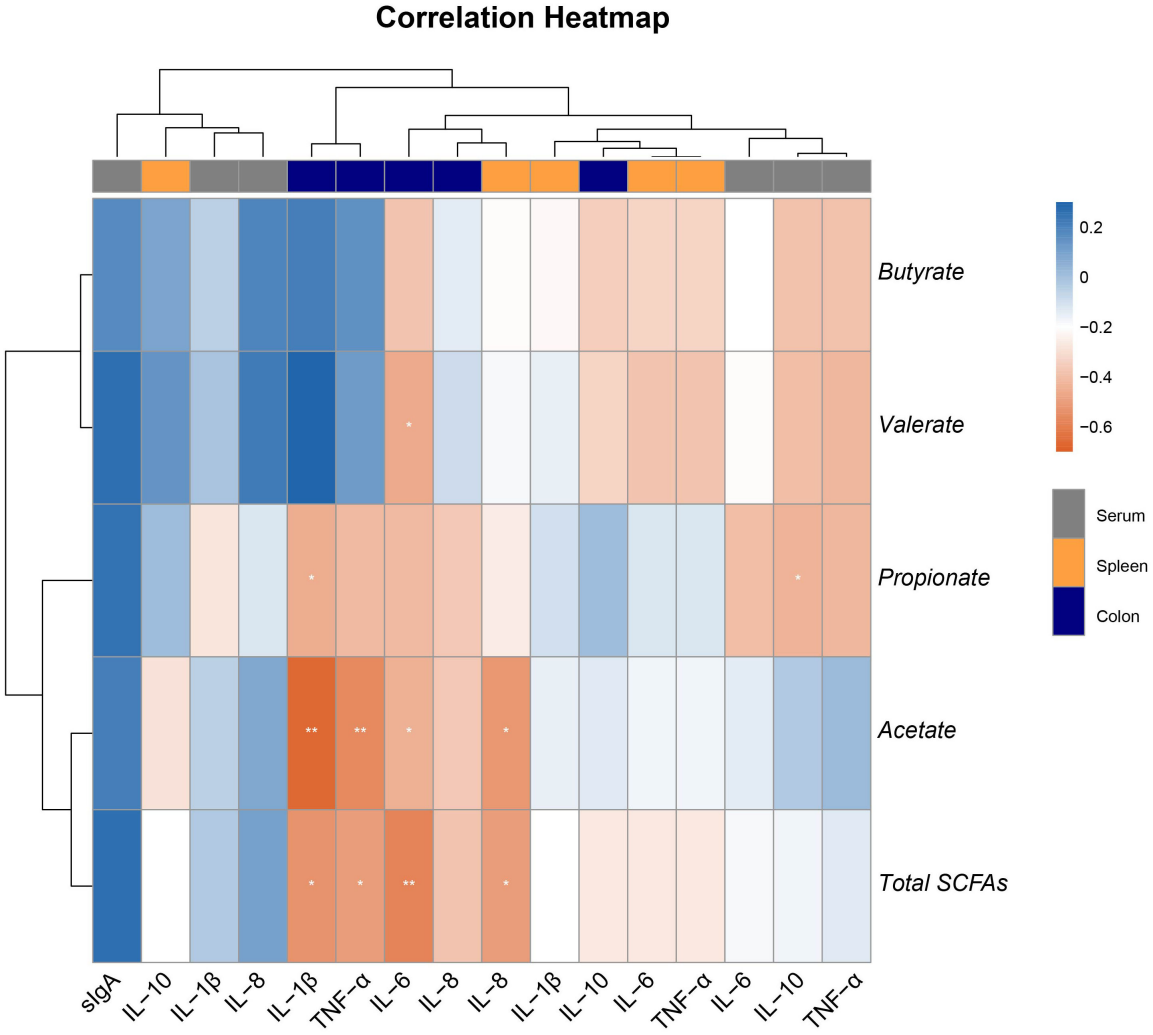
Heatmap of the Spearman’s r correlations between the colonic SCFAs and inflammatory biomarker in serum, spleen, and colon. Data are presented as means ± SEM. * 0.01 ≤ p ≤ 0.05; **0.001 < p ≤ 0.01 (following the correlation analysis).

A Spearman’s correlation was employed to investigate the correlation between inflammatory biomarkers and SCFAs in piglets (**Figure 6**). The results showed that the concentrations of acetate and total SCFAs were significantly negatively correlated with the expression of TNF-α, IL-1β, and IL-6 in the colon, and also significantly negatively correlated with the expression of IL-8 in the spleen (**Figure 6**). Additionally, the concentrations of propionate were significantly negatively correlated with the expression of IL-1β in the colon and IL-10 in serum, while the concentrations of valerate were significantly negatively correlated with the expression of IL-6 in the colon (**Figure 6**). These results suggest that acetate may be the key metabolite in the anti-inflammatory action of Eh.

## 4 Discussion

Due to the increase of drug-resistant bacteria and the ban on feed antibiotics, alternatives need to be found to deal with enterotoxin-producing *Escherichia coli* infections, represented by ETEC K88, which are the main cause of diarrheal diseases in animals, and are particularly harmful to the intestinal tract (Zeyner and Boldt, 2006; Zhang et al., 2022a). Studies have demonstrated that probiotics can promote the establishment of healthy gut microbiota in piglets, improve nutrient digestion and absorption, and enhance intestinal health (Ding et al., 2021; Ma et al., 2022). In this study, piglets infected with ETEC showed significantly reduced ADG and ADFI, with the highest diarrhea rate and rectal temperature. However, oral administration of the probiotic - Eh alleviated the high diarrhea rate and low ADFI induced by ETEC infection, indicating its effectiveness in mitigating growth performance decline and increased diarrhea caused by ETEC.

As a crucial component of the host digestive system, the intestine plays a vital role in food digestion, nutrient absorption, and metabolism (Abdallah et al., 2020; Zhang, 2022). Indicators such as villus height, crypt depth, and the V/C are important measures of digestive and absorptive capacity (Assis et al., 2021; Zhu et al., 2022). ETEC infection can damage the mucosa and the crypt-villus axis, leading to villus atrophy and increased crypt depth, thereby affecting intestinal absorption capacity (Augustino et al., 2022). In this study, ETEC infection significantly reduced jejunal villus length and V/C while increasing crypt depth. Orally Eh significantly increased jejunal villus length and V/C in ETEC-infected piglets and reduced crypt depth. Additionally, the number of goblet cells and crypt depth in the colon of ETEC-infected piglets also increased significantly after probiotic administration. These results indicate that Eh can improve the intestinal morphology of ETEC-infected piglets.

Following ETEC infection, piglets experience initial colonization of the small intestine epithelium, where the bacteria proliferate and secrete enterotoxins, disrupting the intestinal mucosa and increasing intestinal wall permeability, leading to inflammatory responses (Sun et al., 2021; Augustino et al., 2022). Levels of inflammatory cytokines are direct indicators of the host’s inflammatory response, playing a crucial role in regulating immune function and promoting rapid immune reactions following pathogenic infection (Paludan et al., 2021; Sierawska et al., 2022). Moreover, extensive research has confirmed that probiotics possess immunomodulatory effects, enhancing the host’s innate immune system to combat infections and suppress inflammation (Begum et al., 2021; Cristofori et al., 2021). In this study, ETEC infection elevated serum levels of pro-inflammatory factors (TNF-α, IL-1β, IL-6) and expression of intestinal tissue pro-inflammatory factors (TNF-α, IL-1β, IL-6, IL-8). And orally Eh reversed these effects. Using an ETEC-infected IPEC-J2 cell model, we further validated that Eh could improve intestinal inflammation levels. Additionally, we found that orally Eh significantly increased the levels of sIgA in ETEC-infected piglets. SIgA is a major antibody produced by B cells in the intestine, which plays a key role in host immunity (Li et al., 2020; Pabst and Slack, 2020). These results suggest that Eh enhances the host’s anti-inflammatory capacity to counteract immune responses induced by ETEC infection.

The spleen is the vital lymphoid organ an plays a crucial role in responding to oxidative stress and immune defense (Liu et al., 2022). It is also a central organ for nutrient metabolism and detoxification, making it highly susceptible to oxidative stress and inflammatory reactions from various environmental factors (Liu et al., 2022; Wang et al., 2023). Studies have shown that the immune-inflammatory status of the body can be reflected by the spleen index (Yan et al., 2020). In this study, ETEC infection significantly increased the spleen index of piglets and elevated the expression of pro-inflammatory factors such as TNF-α, IL-6, and IL-8. Orally Eh significantly reduced the spleen index and IL-8 expression. This further demonstrates that Eh can mitigate the host inflammatory response induced by ETEC infection, thereby reducing diarrhea.

As the major metabolites of intestinal microbiota, SCFAs provide energy to the body, regulate the intestinal microbiota, inhibit the production of harmful substances in the intestine, and suppress the release of inflammatory factors (Yao et al., 2022; Zhang et al., 2022b). Probiotics can produce large amounts of SCFAs by fermenting carbohydrates in the animal intestine, lowering intestinal pH, inhibiting pathogenic bacteria, and maintaining intestinal health. Research indicates that adding probiotic preparations during the piglet stage can significantly increase lactate and butyrate content in the colon (Li et al., 2021). In this study, Eh significantly increased the content of acetate, propionate, and total SCFAs in the colonic contents of ETEC-infected piglets. Interestingly, we also found that orally Eh in ETEC-infected piglets resulted in a higher proportion of acetate, and the content of acetate in the colon was associated with the expression of inflammatory factors in the colonic and splenic tissues of piglets. This suggests that Eh can alleviate host inflammatory responses by promoting the content of acetate and other SCFAs in the colon of ETEC-infected piglets, thereby reducing diarrhea.

**Figure 7:**
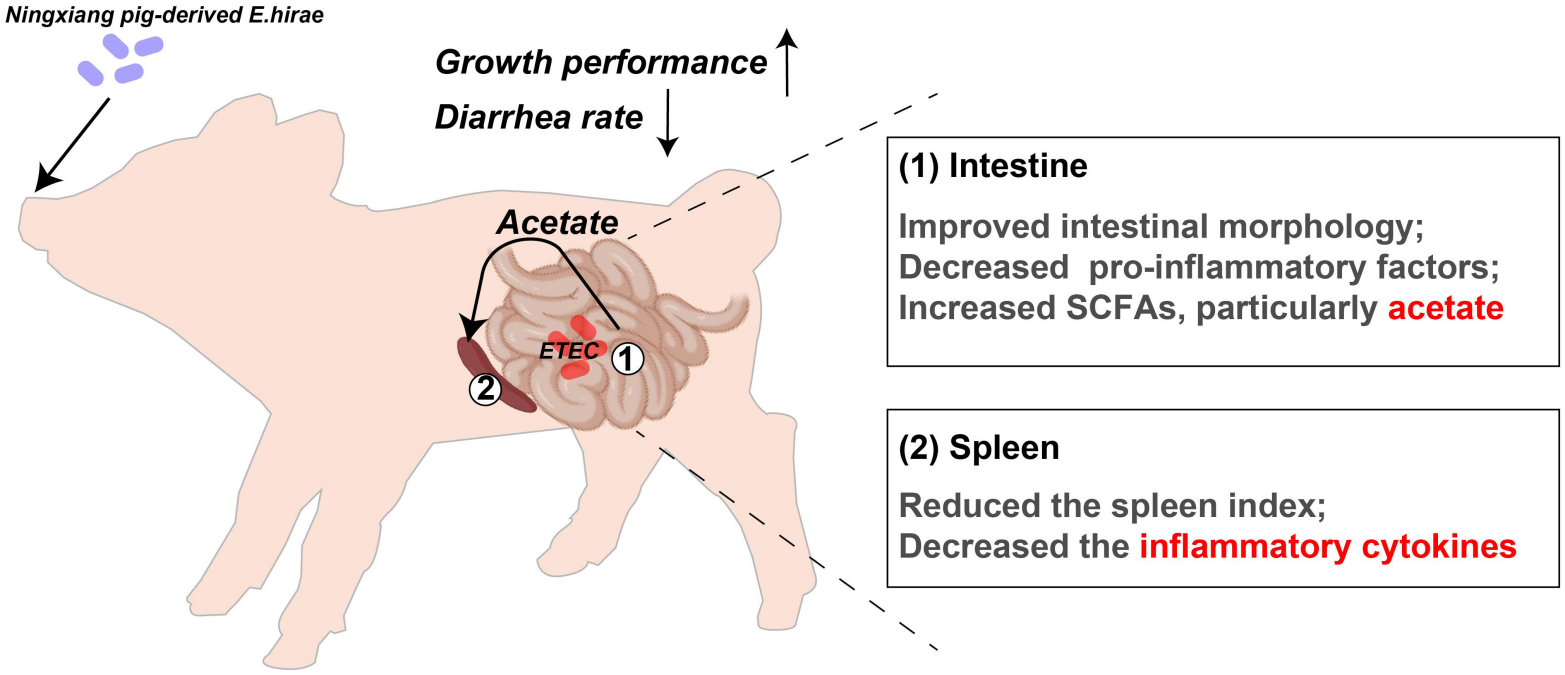
Schematic diagram of *Enterococcus hirae* regulates the inflammatory function and enhances the protection of piglets against ETEC challenge via intestine-acetate-spleen axis.

In conclusion, *Enterococcus hirae* derived from Ningxiang pigs effectively alleviates ETEC-induced diarrhea in weaned piglets, improves their growth performance, and enhances intestinal morphology. Significantly, Eh modulates the intestine-acetate-spleen axis by its regulation of the spleen index and mitigation of inflammatory responses induced by ETEC infection through increased acetate content in colonic chyme (**Figure 7**). This study highlights the intestine-acetate-spleen axis as a critical mechanism and presents Eh as a viable alternative to antibiotics for addressing challenges posed by ETEC K88 infection.

## Data Availability

The original contributions presented in the study are included in the article/**Supplementary Material**. Further inquiries can be directed to the corresponding author.
