# Supplementary material for: Ningxiang pigderived Enterococcus hirae regulates the inflammatory function and enhances the protection of piglets against ETEC challenge
Source: Front Cell Infect Microbiol. 2024 Oct 17;14:1476564. doi: 10.3389/fcimb.2024.1476564 (PMC11525010; doi:10.3389/fcimb.2024.1476564)
Supplement: Supplementary file 1 [file DataSheet1.docx]

**Supplementary Figures**

**
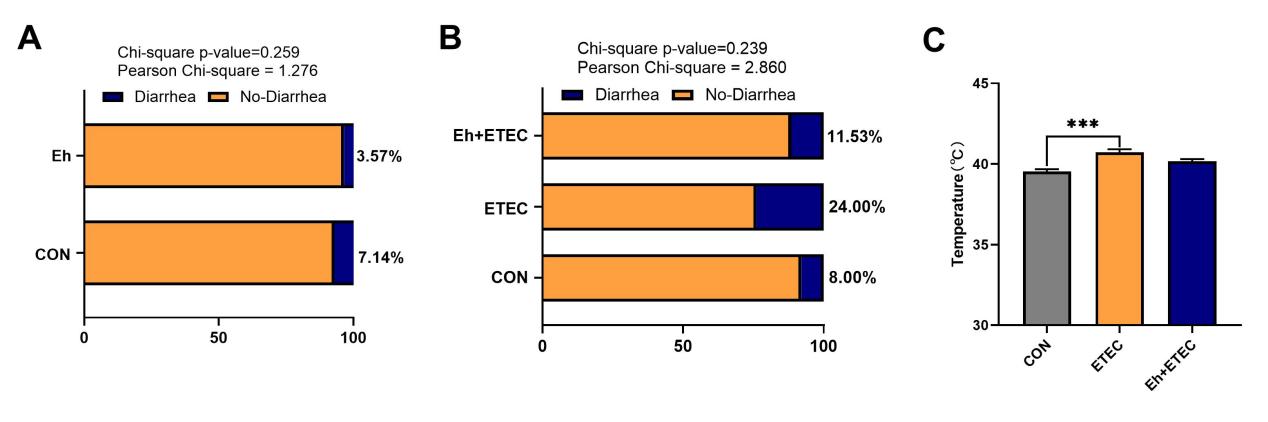
**

**Supplementary Figure S1.** Effects of *Enterococcus hirae* on diarrhea rate and anus temperature in ETEC-challenged Piglets. A, The diarrhea rate of piglets before ETEC challenged. B, The diarrhea rate of piglets after ETEC challenged. C, The anus temperature of piglets. Mean ± SEM are shown. * 0.01 ≤ p ≤ 0.05; **0.001 < p ≤ 0.01 ; *** p ≤ 0.001.

**
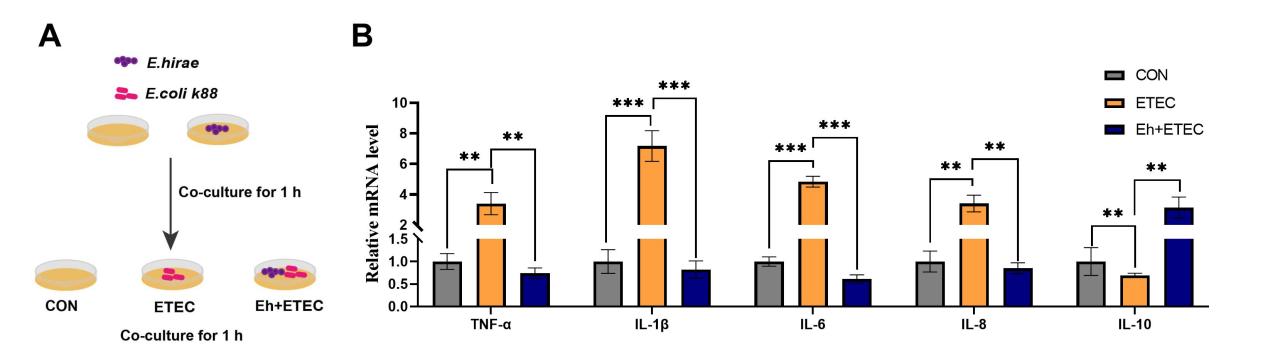
**

**Supplementary Figure S2.** Effects of *Enterococcus hirae* on inflammatory expression in ETEC-challenged IPEC-J2 cells. A, Experimental design diagram. B, The effects of *Enterococcus hirae* in IPEC-J2 cells mRNA inflammatory expression. The Mean ± SEM are shown. **0.001 < p ≤ 0.01 ; *** p ≤ 0.001.

**Supplementary Tables**

**Supplementary Table S1. Basic diet composition and nutritional component (%, as-fed basis)**

| Item | Contents |
| --- | --- |
| **Ingredients** |  |
| Corn | 42.02 |
| Soybean meal | 20.00 |
| [Extruded full-fat soybean](https://www.so.com/link?m=agEAMwkp3L%2F2m9NPkrtD5KN2dX8lapw5iDdQBZMLObR6q6slzoNWOP1lQJAzZ1zXvxGcMSKaaDV9NTx6ywrZcVxUhefg8HNk7dyOx6e9lPN8HBgAKX5NxYy7skngO00XJUwKpgJbBnMRi2MqmRbLLsA2CALbtb3N0XhN4e1aE5FcadTck8F8Nzv4c%2F7VNClFaOmYperVF%2BUew%2B8ifyQ92fOyqKqTuxToYYWLvCNKZ9Cvlo%2FBFI8tmHUyjItDMQ5SSyPbl7itshcUjgMQHzFPf7zjmMqWwKNVxWAnmgg%3D%3D) | 12.00 |
| Soy protein concentrate | 4.00 |
| Fermented cottonseed meal | 0.00 |
| Whey powder | 8.00 |
| Soy oil | 4.61 |
| Sucrose | 5.38 |
| Dicalcium phosphate | 1.26 |
| Limestone | 0.99 |
| Salt | 0.30 |
| Lysine | 0.41 |
| Methionine | 0.13 |
| Threonine | 0.13 |
| Tryptophan | 0.02 |
| Chromic oxide | 0.25 |
| Vitamin-mineral premix^1^, no antibiotic | 0.50 |
| **Total** | 100.00 |
| **Nutrient levels**^2^ |  |
| Metabolized energy, kcal/kg | 3.40 |
| Digestible energy, Mcal/kg | 3.47 |
| Dry matter | 87.60 |
| Crude protein | 21.13 |
| Total calcium | 0.63 |
| Total phosphorus | 0.65 |
| Total lysine | 1.51 |
| Total threonine | 0.94 |
| Total tryptophan | 0.40 |
| Total methionine + cystine | 0.86 |

^1^ The components and contents of the premix providing nutrients for per kg feed are as follows: Vitamin A, 12,000 IU; Vitamin D3, 2500 IU; Vitamin E, 30 IU; Vitamin K3, 30 mg; Vitamin B12, 12 micrograms; Riboflavin, 4 mg; Pantothenic acid, 15 mg; Niacin, 40 mg; Choline chloride, 400 mg; Folic acid, 0.7 mg; Vitamin B1, 1.5 mg; Vitamin B6, 3 mg; Biotin, 0.1 mg; Manganese, 40 mg; Iron, 90 mg; Zinc, 100 mg; Copper, 8.8 mg; Iodine, 0.35 mg; Selenium, 0.3 mg.

^2^All nutrient levels were analyzed values, except digestible energy and metabolizable energy.

**Supplementary Table S2. Primers used for gene expression analysis through Real-Time PCR.**

| Genes | Forward | | Reverse |
| --- | --- | --- | --- |
| *PCNA* | | TACGCTAAGGGCAGAAGATAATG | CTGAGATCTCGGCATATACGTG |
| *IL-6* | | ATCCTCGACGGCATCTC | TCAGCCATCTTTGGAAGG |
| *TNF-α* | | TGTGTGGCTGCAGGAAGAAC | GCAATTGAAGCACTGGAAAAGG |
| *IL-8* | | GACATACTCCAAACCTTTCCA | AACTTCTCCACAACCCTCTG |
| *IL-10* | | CTGCATCCACTTCCCAACCA | AGAAACTCTTCACTGGGCCG |
| *IL-1β* | | AACGTGCAGTCTATGGAGT | GAACACCACTTCTCTCTTCA |
| *β-actin* | | GCGTAGCATTTGCTGCATGA | GCGTGTGTGTAACTAGGGGT |
